# Immunogenicity and Effectiveness of Primary and Booster Vaccine Combination Strategies during Periods of SARS-CoV-2 Delta and Omicron Variants

**DOI:** 10.3390/vaccines10101596

**Published:** 2022-09-22

**Authors:** Rima Moghnieh, Claude El Hajj, Dania Abdallah, Nayla Jbeily, Abdul Rahman Bizri, Mohamed H. Sayegh

**Affiliations:** 1 Division of Infectious Diseases, Department of Internal Medicine, Makassed General Hospital, Beirut P.O. Box 11-6301, Lebanon; 2 Faculty of Medicine, Lebanese University, Beirut P.O. Box 6573/14, Lebanon; 3 Middle East Airlines-AIRLIBAN S.A.L., Beirut P.O. Box 206, Lebanon; 4 Pharmacy Department, Makassed General Hospital, Beirut P.O. Box 11-6301, Lebanon; 5 FMPS Holding S.A.L., Beirut P.O. Box 60 247, Lebanon; 6 Department of Internal Medicine, Division of Infectious Diseases, American University of Beirut Medical Center, Beirut P.O. Box 11-0236, Lebanon; 7 GAP Solutions under Contract No. 75N93019D00026 with National Institute of Allergy and Infectious Diseases, National Institutes of Health, Department of Health and Human Services, United States of America, Washington, DC 20201, USA; 8 Faculty of Medicine, American University of Beirut, Beirut P.O. Box 11-0236, Lebanon

**Keywords:** effectiveness, immunogenicity, homologous vaccination, heterologous vaccination, Gam-COVID-Vac, BNT162b2, Delta variant, Omicron variant, Lebanon

## Abstract

In this study involving a cohort of employees of the National Airline company in Lebanon, we assessed humoral immunity levels and the effectiveness of two COVID-19 vaccines, Gam-COVID-Vac versus BNT162b2, after two doses and after a homologous and heterologous BNT162b2 booster, in addition to the impact of hybrid immunity. Vaccine effectiveness (VE) was retrospectively determined against laboratory-confirmed SARS-CoV-2 infection during the periods of Delta and Omicron variants’ predominance, separately, and was calculated based on a case–control study design. The humoral immune response, measured by a SARS-CoV-2 anti-spike receptor-binding domain (RBD) IgG titer, was prospectively assessed after the aforementioned vaccination schemes at different time points. This study showed higher effectiveness of BNT162b2 after two doses (81%) compared to two doses of Gam-COVID-Vac (41.8%) against the Delta variant of SARS-CoV-2, which correlated with anti-spike antibody levels. Regarding the Omicron variant, protection against infection and antibody levels were severely compromised and the correlation between an anti-spike IgG titer and effectiveness was lost, unlike the situation during the Delta wave. Considering the booster vaccination schemes, a homologous BNT162b2 booster after a BNT162b2 primary vaccination induced a higher humoral immune response when compared to that induced by a heterologous BNT162b2 booster after a Gam-COVID-Vac primary vaccination. However, the VE of both booster regimens against the Omicron variant was almost equal (64% in the homologous regimen and 57% in heterologous regimen). Hybrid immunity evidenced a better humoral response and a greater and longer protection against Delta and Omicron infections compared to vaccination-induced immunity in COVID-19-naïve individuals. Finally, the findings show that VE waned with time during the same wave, highlighting the importance of reinforcing primary and booster COVID-19 vaccination mainly at the beginning of each wave during the surge of a new variant of concern.

## 1. Introduction

The historical success story of smallpox eradication achieved through active global immunization campaigns has been the most efficient strategic approach in the fight against vaccine-preventable infectious diseases [1]. This milestone is far from reach for SARS-CoV-2, despite the massive efforts made by researchers, public health officials, and companies in developing, approving, producing, and delivering COVID-19 vaccines all over the globe.

Factors delaying the progress of global vaccination coverage include vaccine hesitancy, the global shortage of vaccine supply, and inequitable vaccine distribution, especially among low- and middle-income countries [2,3]. Other factors are related to the type of the vaccine itself and its ability to induce an effective immune response, along with its duration of protection [4,5,6]. Another element to consider is that continuously mutating RNA viruses such as SARS-CoV-2 have the ability to undergo an antigenic evolution, compared to the relatively stable DNA viruses [7,8]. Partial or full vaccine evasion are more likely to be caused by these mutations and thus cause breakthrough infections, leading to reduced effectiveness with newly appearing variants [8].

One of the first vaccines that was granted emergency use authorization by regulators in their countries of origin was the Gamaleya Sputnik V adenoviral vector vaccine (Gam-COVID-Vac) [9,10]. The Gamaleya Sputnik V received full permanent approval from Russia’s Ministry of Health in February 2022 [11]. Around 70 countries have used Gam-COVID-Vac in their primary vaccination protocols, not to mention that multiple facilities were contracted to manufacture Gam-COVID-Vac. It is distributed in 17 countries around the world [12,13]. Data on several aspects were needed to orient policymakers a few months after primary Gam-COVID-Vac vaccination and after the emergence of the relatively vaccine-tolerant Omicron variant: (1) whether a booster vaccine was needed, (2) whether the booster vaccine was effective against breakthrough infections, (3) whether a safe option is present or not, and (4) what was the adequate booster vaccination timing.

Real-world effectiveness data on primary Gam-COVID-Vac vaccination were reported from Latin America, namely, from Mexico [14]; however, no data are available on a heterologous booster after Gam-COVID-Vac primary vaccination. Fortuitously, a successful and safe experience was previously established with a heterologous BNT162b2 booster in individuals primed with ChAdOx1 nCoV-19 (AZD1222). This is another adenoviral vector vaccine [15,16,17], which paved the way for heterologous booster regimens post Gam-COVID-V priming.

In Lebanon, COVID-19 vaccines of different platforms were granted emergency use authorization (EUA) from the Lebanese Ministry of Health (MOH), one of which was Gam-COVID-Vac, which received EUA in April 2021. The different availability of vaccines from different platforms presented an opportunity to compare the immunogenicity and effectiveness of these vaccines, as well as the safety and immunogenicity of their booster protocols.

Herein, we conducted a case–control study to determine the effectiveness of Gam-COVID-Vac and BNT162b2 primary vaccination regimens against two variants of concern predominantly circulating in Lebanon at different points in time, namely, the Delta and Omicron variants. We also studied the effectiveness of a BNT162b2 booster dose after primary BNT162b2 and Gam-COVID-Vac vaccination against COVID-19 during the Omicron variant’s predominant phase. The booster BNT162b2 dose’s safety was also assessed in individuals previously vaccinated with Gam-COVID-Vac. We assessed the impact of hybrid immunity on VE across the previously mentioned regimens during the Delta and Omicron waves. We hypothesized that the extent of protection against these variants provided by previous infection and vaccination was superior to that provided by vaccination alone. On the other hand, we measured SARS-CoV-2 anti-spike receptor-binding domain (RBD) immunoglobulin G (anti-S-IgG) titers, as a marker of the immunogenicity of the studied vaccine regimens. We hypothesized that the positive correlation between the levels of anti-S-IgG detected after a homologous or heterologous BNT162b2 booster and the protection against Omicron infection is lost.

## 2. Materials and Methods

### 2.1. Study Population, Data Sources, and Study Design

This study is divided into three parts:

- A retrospective part dealing with the different homologous and heterologous vaccination schemes’ effectiveness at different time points.
- A prospective part dealing with the different homologous and heterologous vaccination schemes’ immunogenicity at different time points.
- A retrospective part dealing with the safety of heterologous booster vaccination with one BNT162b2 dose after primary two-dose Gam-COVID-Vac vaccination.

#### 2.1.1. Vaccine Effectiveness (VE)

This is a retrospective, cohort, case–control study that examined the effectiveness of two-dose primary vaccination regimens (2*Gam-COVID-Vac or 2*BNT162b2) and homologous (2*BNT162b2/1* BNT162b2) or heterologous (2*Gam-COVID-Vac/1BNT162b2) booster vaccination regimens against laboratory-confirmed SARS-CoV-2 infections during the Delta and Omicron surges versus the control, i.e., unvaccinated employees.

The study population included an employee cohort at the National Airline Company [Middle East Airlines (MEA)] in Beirut, Lebanon. This company was established in 1945 and owns five subsidiaries. The company has an onsite clinic with a registry of all health-related information, including demographic characteristics, chronic health conditions, SARS-CoV-2 PCR tests, COVID-19 vaccination history, and reasons for absences.

MEA employees of the main company and its subsidiaries were offered a two-dose primary vaccination series with either Gam-COVID-Vac (available for the private sector) or BNT162b2 during the COVID-19 vaccination campaign launched by the company in April 2021. The company decided to offer its employees a BNT162b2 booster dose 6 months after primary vaccination with Gam-COVID-Vac or BNT162b2 schemes, starting October 2021.

An email was sent by the human resources department at MEA to all employees explaining its aims and requesting their consent to participate in the VE analyses by sharing their health-related data prior to the study launch, while ensuring anonymity. 

Employees from all age groups and from both genders were included. Those who were on renal replacement therapy or had any history of congenital or acquired immunosuppression (including asplenia, congenital or acquired immunodeficiency, solid/hematologic malignancy or disease treated by chemotherapy or immunosuppressive therapy, solid organ transplant, or long-term systemic corticosteroids and radiation therapy) were excluded. Employees who received vaccination schemes other than what was formerly mentioned were excluded from the analysis.

Data, including SARS-CoV-2 PCR testing, COVID-19 vaccination history, clinical infection data, and other related demographic details from the start of the pandemic, were extracted from the digital institutional health information database based on the employee medical records at the company clinic. Since the start of the pandemic, the company’s administration followed the US Centers for Disease Control and Prevention’s (CDC) COVID-19 recommendations and guidelines regarding SARS-CoV-2 PCR testing, contact tracing, isolation and quarantine, and absenteeism. SARS-CoV-2 PCR testing was done based on clinical suspicion due to presence of symptoms compatible with a respiratory tract infection or suspicion of infection after contact tracing. Testing during contact tracing was performed irrespective of whether the index case was at work or at home. Daily symptom checking was done by supervisors of each department along with a daily survey about any risk of exposure to suspected or confirmed COVID-19 cases. All employees were educated about COVID-19 clinical manifestations and its high contagiousness and rapid spread.

A PCR-positive swab, regardless of the reason for PCR testing or the presence of symptoms, was used to define laboratory-confirmed SARS-CoV-2 infections. Breakthrough SARS-CoV-2 infections were considered at least 14 days after the second dose of primary vaccination with Gam-COVID-Vac or BNT162b2 and at least 7 days after receipt of the BNT162b2 booster dose [18,19]. A positive SARS-CoV-2 PCR test within 3 weeks of a previous positive SARS-CoV-2 PCR test was dismissed and not counted as a new infection.

In Lebanon, the pandemic was defined by the dominance of four SARS-CoV-2 lineages, upon analyzing the whole Lebanese dataset in GISAID [20,21].The genomes available in GISAID show that, by July of 2021, the variant of concern, Delta (B.1.617.2), had already completely substituted Alpha and was, in turn, completely replaced by Omicron (sublineages BA.1 and BA.1.1) by the end of December 2021 [20,21]. Coincidently, the appearances of both lineages (Delta and Omicron) were followed by new surges in the number of cases in Lebanon [20,21]. Accordingly, employees were referred to as SARS-CoV-2 Delta cases if they first tested positive between 1 August 2021, and 20 December 2021, as the majority of the cases in Lebanon were predominantly attributable to the circulating Delta variant during that time [20,21]. Employees were referred to as SARS-CoV-2 Omicron (B.1.1.529) cases if they first tested positive between 1 January 2022, and 28 February 2022, as the Omicron variant was the predominant circulating variant during that period [20]. Both symptomatic and asymptomatic infections were considered. Participants testing positive were only included before 1 March 2022, as this was the end of the Omicron study period. Cases between 21 December 2021, and 31 December 2021, were not included, as it was considered a washout period, and to minimize the residual Delta infection occurrence incidence in the community at the beginning of the Omicron wave.

VE analyses were stratified according to primary immunization schemes (Gam-COVID-Vac or BNT162b2 vaccine). VE was assessed for each primary course in intervals of <3 months, 3 to <6 months, and ≥6 months after the second dose. Heterologous BNT162b2 booster schedules were assessed <3 months after the booster dose. VE was calculated for each vaccination scheme (1) including all participants with and without a previous COVID-19 exposure status (i.e., before Delta and Omicron surges, separately), (2) in COVID-naïve participants, and (3) in those with hybrid immunity (with a COVID-19 exposure history and recovery either before the primary or booster vaccination before the Delta and Omicron surges, separately).

Ethics approval was obtained from MGH’s Institutional Clinical Research Ethics Committee (approval number: 892021).

#### 2.1.2. Humoral Immunity

Immunogenicity assessment at several time points in the subgroup analyses was prospectively done through a digital random employee recruitment from MEA: those who previously received Gam-COVID-Vac and those who received BNT162b2 vaccines. Participants were assigned to different groups according to the primary vaccination type and whether they received a booster dose. Classification is as follows:

1. At <3 months after two BNT162b2 doses: employees received two BNT162b2 doses (21 days apart) in <3 months prior to the time of assessment.
2. At 3 to <6 months after two BNT162b2 doses: employees received two BNT162b2 doses (21 days apart) in the past 3 months up to <6 months prior to the time of assessment.
3. At 6 months and above after two BNT162b2 doses: employees received two BNT162b2 doses (21 days apart) in the past 6 months or more prior to the time of assessment.
4. At <3 months after one BNT162b2 booster dose following 2*BNT162b2 doses: employees received a single BNT162b2 booster dose after being primarily immunized with two BNT162b2 doses in the past 6 months (booster given at <3 months prior to the time of assessment).
5. At 3 to <6 months after two Gam-COVID-Vac doses: employees received two Gam-COVID-Vac doses (21 days apart) in the past 3 to <6 months prior to the time of assessment. Of note, none of the employees received Gam-COVID-Vac with the second dose given in <3 months prior to the time of assessment. No employees were recruited for the assessment of humoral immunity at >6 months of the second dose.
6. At <3 months after one BNT162b2 booster dose following two Gam-COVID-Vac doses: employees received a single BNT162b2 booster dose after being primarily immunized with two Gam-COVID-Vac doses in the past 6 months (booster given at <3 months prior to the time of assessment).

The number of participants in these groups was dictated by the number of employees that fell into the temporal definitions of the groups. Participants herein were stratified by age, gender, and previous COVID-19 history at any time. Among the primary vaccination schemes, employees who previously received any other type of COVID-19 vaccine than that specified were excluded. Participants in the immunogenicity subgroup analyses provided their written informed consent for health information and blood sample collection.

#### 2.1.3. Sample Collection and Humoral Immunity Measurement

Blood samples were collected from participants in all groups at the MEA clinic and sent to MGH to determine SARS-CoV-2 anti-S-IgG titers measured by immunoassay. Sample analysis was run at the MGH Microbiology Laboratory. The chemiluminescence enzyme immunoassay (Elecsys Anti-SARS-CoV-2 S assay) was used to analyze the antigen-specific humoral immune response, and assay results were measured on the Cobas e 601 immunoassay analyzer (Roche Diagnostics, Basel, Switzerland), with a measuring range from 0.4 U/mL to 250 U/mL (up to 25,000 U/mL with manual 1:100 dilution, and up to 50,000 U/mL with manual 1:200 dilution) [22]. All samples were processed according to the manufacturer’s instructions. Measurement results were in U/mL, with the cut-off point defined as 0.80 U/mL to differentiate samples as reactive (≥0.80 U/mL) and non-reactive (<0.80 U/mL) for anti-S-IgG [22]. The assigned U/mL were equivalent to Binding Antibody Units (BAU)/mL, as defined by the first WHO International Standard for anti-SARS-CoV-2 immunoglobulin (NIBSC code 20/136). Therefore, no conversion of units was required. In all groups, an anti-S-IgG geometric mean titer (GMT) was calculated for the whole group, among vaccinated COVID-19-naïve employees, and among those with hybrid immunity.

## 3. Safety of BNT162b2 Booster Dose in Employees Previously Vaccinated with 2*Gam-COVID-Vac Doses

Safety data consisted of solicited local and systemic adverse events (AE) that occurred 14 days after the booster vaccination in these participants. These data were collected through telemedicine at day 14 of each individual booster by a registered nurse at the MEA clinic, according to a prepared checklist [22].

### Statistical Analysis

Sociodemographic VE analysis cohort characteristics, including age category, gender, and COVID-19 infection/recovery history before each variant surge, were compared between vaccinated and unvaccinated groups using counts and percentages. Categorical analyses on gender and age were performed using the chi-square test.

Multivariable logistic regression was used with the laboratory-confirmed SARS-CoV-2 infections as the dependent variable and case participants being those testing positive (stratified in separate analyses as being infected with either the Omicron or Delta variant, separately). Controls included employees with no SARS-CoV-2 PCR-positive test record during the study period, either due to absence of clinical suspicion or any possibility of contracting COVID-19 based on the vigilant contact-tracing system put in place by the airline company from the start of the pandemic, and irrespective of their vaccination status.

Vaccination status was included as an independent variable, and the effectiveness of different vaccination schemes over individual time periods during the Delta and Omicron waves and the associated 95% CI were calculated by applying the following equation: VE = [1 − adjusted odds ratio (aOR) of vaccination among cases compared with controls] × 100. VE was adjusted in logistic regression models for age (<50, 50–65, >65 years), gender (male, female), and previous history of SARS-CoV-2 infection before the Delta and Omicron surges, individually. These factors were all considered potential confounders and were included in all models.

Using multivariate logistic regression, VE was calculated in the entire cohort with the reference group for all estimates being the unvaccinated employees regardless of previous COVID-19 exposure before the Delta and Omicron surges, separately. Regarding the VE analyses among the COVID-19-naïve subgroup, they were the reference group for all estimates, including persons with no previous infection and no vaccination. The reference group included unvaccinated COVID-19-naïve employees when it was calculated among employees with hybrid immunity (previous infection and vaccination).

Only those vaccinated in this specific time-since-vaccination stratum and those unvaccinated in each VE analysis for a specific time-since-vaccination stratum were included. Accordingly, the number of cases and controls varied across time-since-vaccination analyses. Effectiveness after the second dose was estimated during each month of the Delta wave during the Delta surge, where 1 month was defined as 30 days.

Categorical analyses of gender and age, presented as number and percentage, were performed using the chi-square test for immunogenicity subgroup analysis of different vaccination schemes at different time points among the selected participants. Antibody titers were presented as geometric mean and 95% CI. The one-sample Kolmogorov–Smirnov test was used to check for data distribution normality. The “Kruskal–Wallis” test, followed by Dunn’s multiple comparison post hoc test, was performed to compare unpaired nonparametric data between the groups (antibody levels).

Statistical significance was defined as *p* < 0.05. The IBM Statistical Package for the Social Sciences program for Windows (version 23.0) (Armonk, NY, USA: IBM Corp.) and GraphPad Prism 9.0 software (GraphPad Software, Inc., San Diego, CA, USA) were used to carry out analysis using two-tailed tests.

## 4. Results

### 4.1. Study Population for VE Analyses

**Figure 1:**
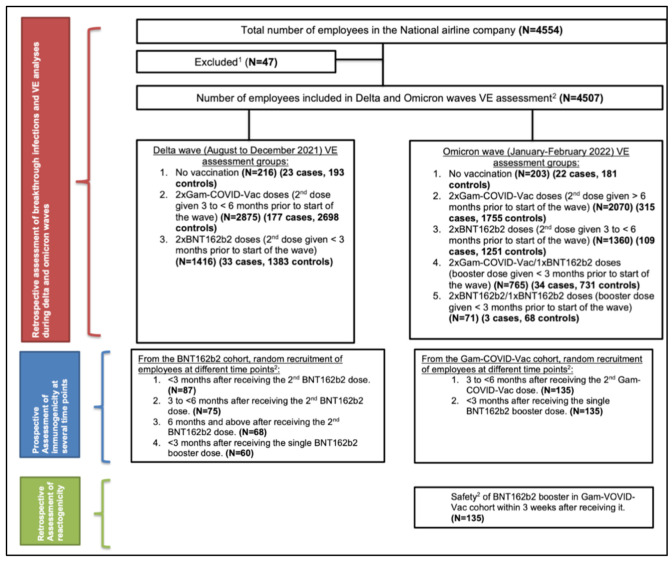
Study profile. VE: vaccine effectiveness. N.B.: ^1^ Employees who were on renal replacement therapy or had any history of congenital or acquired immunosuppression (including asplenia, congenital or acquired immunodeficiency, solid/hematologic malignancy or disease on chemotherapy or immunosuppressive therapy, solid organ transplant, or long-term systemic corticosteroids and radiation therapy) were excluded. Employees who received vaccines other than BNT162b2 or Gam-COVID-Vac were excluded from the analysis. ^2^ Employees from all age groups and from both genders were included in vaccine effectiveness, immunogenicity and safety analyses.

The study profile is illustrated in Figure 1. Airline staff members and its subsidiaries who agreed to participate in this study comprise 4554 employees. A total of 47 participants were excluded from the VE analysis, as per the formerly mentioned exclusion criteria.

#### 4.1.1. Delta Surge Period

In total, data from 4507 MEA employees were included for VE analyses during the Delta surge (from 1 August 2021 to 20 December 2021), including COVID-19-naïve employees and individuals with previous exposure to COVID-19 prior to the Delta variant.

A total of 2875 (63.8%) individuals received two Gam-COVID-Vac doses and the timing of the second dose was between April and May 2021, by August 2021 (start of the Delta wave), which corresponded to 3 to <6 months before the start of the Delta wave.

Meanwhile, 1416 (31.4%) individuals received two BNT162b2 doses and the timing of the second dose was between July and August 2021, which corresponded to <3 months before the start of the Delta wave.

A total of 216 (4.8%) individuals remained unvaccinated during the Delta wave.

None of the employees received a third BNT162b2 shot during this period.

The majority of the study participants were under 50 years of age (74.1%), and approximately 73.4% were males. This age and gender distribution was comparable among all vaccination groups (*p* < 0.0001). The demographic characteristics of the study population and previous COVID-19 exposure status prior to this period are presented in Supplementary Table S1.

#### 4.1.2. Omicron Surge Period

In total, data from 4469 MEA employees were included for VE analyses during the Omicron surge [from 1 January 2022 to 28 February 2022 (end of study)], including COVID-19-naïve employees and individuals with previous COVID-19 exposure prior to the Omicron variant (Figure 1).

Herein, 2070 (46.3%) individuals were already vaccinated with two Gam-COVID-Vac doses between April and May 2021, which corresponded to ≥6 months before the start of the Omicron wave, and 765 (17.1%) individuals received a BNT162b2 booster dose after two Gam-COVID-Vac doses, and within <3 months of the start of the wave.

Meanwhile, 1360 (30.4%) individuals were already vaccinated with two BNT162b2 doses between July and August 2021, which corresponded to 3 to <6 months before the start of the Omicron wave, and 71 individuals received a third BNT162b2 dose 6 months after two BNT162b2 doses, and within <3 months of the start of the wave.

However, 203 (4.5%) individuals remained unvaccinated during the Omicron wave.

Over this timeframe, the majority of the participants were under 50 years of age (74.4%), and approximately 73.3% were males. This age and gender distribution was comparable among all vaccination groups (*p* < 0.0001). The study population demographic characteristics, as well as previous COVID-19 exposure status before this period, are presented in Supplementary Table S2.

### 4.2. VE against Delta Variant

#### 4.2.1. VE among All Participants, including Those with and Those without Previous Exposure to COVID-19 Prior to the Delta Wave

During the Delta wave, two homologous vaccination schemes were available for evaluation involving Gam-COVID-Vac, with its second dose given at 3 to <6 months prior to the start of the wave, and BNT162b2, with its second dose given at <3 months prior to the start of the wave. None of the employees received Gam-COVID-Vac within <3 months of the start of the Delta wave. VE results during the Delta wave are shown in Table 1.

In the unvaccinated population, 23/216 participants developed SARS-CoV-2 Delta infections (10.7%), among whom one case was hospitalized and no deaths were recorded.

A total of 177 SARS-CoV-2 breakthrough infections were recorded among participants who received two Gam-COVID-Vac doses (177/2875, 6.2%), given within 3 to <6 months of the start of the Delta wave. Accordingly, Gam-COVID-Vac effectiveness reached 41.8% (95% CI, 5.6–62.6). From this group, one case was hospitalized, yet did not progress to critical or fatal COVID-19.

Meanwhile, 33 SARS-CoV-2 breakthrough infections were recorded among all participants who received two BNT162b2 doses (33/1416, 2.3%), given within <3 months of the start of the Delta wave. BNT162b2 effectiveness during this period reached 81.0% (95% CI, 66.3–89.2). None of the breakthrough infections in this group progressed to severe, critical, or fatal COVID-19.

#### 4.2.2. Longitudinal Variation in the Effectiveness of the Different Vaccination Schemes in Subsequent Months during the Delta Wave

**Figure 2:**
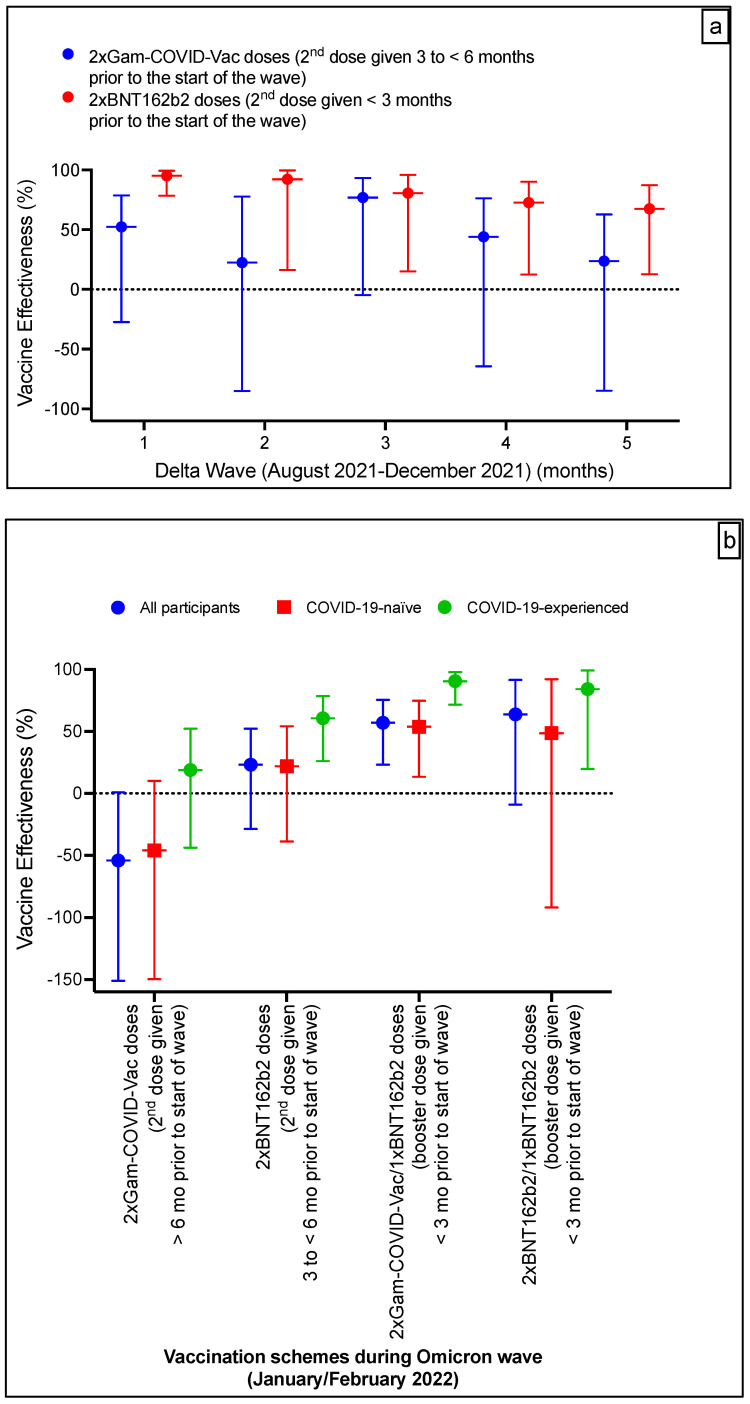
Effectiveness of the available COVID-19 vaccination schemes during the Delta wave (**a**) and Omicron Wave (**b**). N.B. Data are presented as effectiveness point estimates, with error bars indicating the corresponding 95% confidence intervals.

A gradual waning was observed in the effectiveness of the available vaccination schemes (Table 1 and Figure 2a) during the Delta wave (5 months). Gam-COVID-Vac effectiveness was 52.3% (95% CI, −27.4–78.7) at 3 to <6months after the second dose during the wave’s first month. This effectiveness declined with time to reach 23.7% (95% CI, −84.7–62.8) by the end of the wave (>6 months after the second dose; fifth month of the wave). BNT162b2 effectiveness was highest at 95.1% (95% CI, 78.4–99.3) at <3 months after the second dose during the first month of the wave, on the other hand. This effectiveness declined with time to reach 67.5% (95% CI, 12.8–87.2) by the end of the wave (3 to <6 months after the second dose; fifth month of the wave). For comparison, Gam-COVID-Vac effectiveness against any Delta infection was lower than that of BNT162b2 (average VE, 50.5% vs. 73.6%, respectively) during the same timeframe from the second dose (at 3 to <6 months).

### 4.3. VE against Omicron Variant among All Participants, including Those with and without Previous COVID-19 Exposure

During the Omicron wave, two homologous vaccination schemes were available for evaluation involving Gam-COVID-Vac, with its second dose given at ≥6 months prior to the start of the Omicron wave, and BNT162b2, with its second dose given at 3 to <6 months prior to the start of the wave. Booster BNT162b2 schemes among the primary Gam-COVID-Vac or BNT162b2 vaccination were available for VE analyses, with the booster given at <3 months of the wave’s start. VE results are shown in Table 2 and Figure 2b.

In the unvaccinated population, 22/203 participants developed SARS-CoV-2 during the Omicron phase until the study period’s end (10.8%).

A total of 315 Omicron breakthrough infections were recorded among participants who received two Gam-COVID-Vac doses (315/2070, 15.2%), given ≥6 months before the start of the Omicron wave. No protective vaccination effect against infection caused by the Omicron variant was noted (VE = −54.1%, [95% CI, −151–0.84]), compared with no vaccination in this primary Gam-COVID-Vac cohort.

Among individuals who received a BNT162b2 booster dose after two Gam-COVID-Vac doses, 34/765 (4.4%) developed breakthrough infections, with the booster dose given within <3 months of the Omicron wave start. Accordingly, effectiveness against Omicron infection rebounded to 57% (95% CI, 23.2–75.5) within <3 months of giving the BNT162b2 booster dose in this group.

Meanwhile, 109 SARS-CoV-2 breakthrough infections were recorded among all participants who received two BNT162b2 doses (109/1360, 8.0%), given within 3 to <6 months of the start of the Omicron wave. Hence, primary BNT162b2 effectiveness against Omicron variant infection in this case was 23.1% (95% CI, −28.7–52.1).

Omicron breakthrough infections were recorded among 3/71 individuals who received a third BNT162b2 dose after two BNT162b2 doses (4.2%), with the booster dose given within <3 months of the start of the Omicron wave. Consequently, effectiveness against Omicron infection rebounded to 63.7% (95% CI, −9.1–91.6) within <3 months of giving a BNT162b2 booster dose in this group.

For comparison, Gam-COVID-Vac was not protective against any Omicron infection, compared to BNT162b2 (VE, −54.1% vs. 23.1%, respectively), during the same time frame from the second vaccine dose, i.e., at 6 months and beyond. However, after a BNT162b2 booter in both groups, VE in the two BNT162b2 and one BNT162b2 groups was marginally higher than that of the two Gam-COVID-Vac and one BNT162b2 groups (63.7% vs. 57%, respectively).

None of the Omicron infections in any of the aforementioned groups progressed to severe, critical, or fatal COVID-19.

### 4.4. Effectiveness of COVID-19 Vaccination Alone versus Hybrid Immunity against Delta and Omicron Variants

Month-by-month VE calculation was not possible in each of the corresponding COVID-19-naïve or COVID-19-experienced vaccination groups during the Delta wave. It could be calculated only by the end of the wave during December 2021 (Table 1).

The effectiveness of two Gam-COVID-Vac doses, with the second dose given at >6 months by the end of the Delta wave in COVID-19-naïve participants, was 20.1% (95% CI, −94.7–61.6). However, the effectiveness of hybrid immunity (previous infection and two Gam-COVID-Vac doses given at >6 months) was much higher, reaching 82.6% (95% CI, 44.4–94.9) (Table 1).

On the other hand, the effectiveness of two BNT162b2 doses given at 3 to <6 months by the end of the Delta wave in COVID-19-naïve participants was 71.8% (95% CI, 22.3–89.1) and that of hybrid immunity (previous infection and two BNT162b2 doses given at 3 to <6 months by the end of the wave) was 88.1% (95% CI, 56.5–97.5) (Table 1).

The effectiveness of two Gam-COVID-Vac doses given at >6 months prior to the start of the wave in COVID-19-naïve participants was not protective (−46%; 95% CI, −149–10.1) (Table 2, Figure 2b) during the Omicron wave. However, the effectiveness of hybrid immunity (previous infection and two Gam-COVID-Vac doses given at >6 months prior to the start of the wave) was higher, reaching 18.8% (95% CI, −43.8–52.2).

The effectiveness of two BNT162b2 doses given at 3 to <6 months prior to the start of the Omicron wave in COVID-19-naïve participants was 21.9% (95% CI, −38.9–54.1) and that of hybrid immunity (previous infection and two BNT162b2 doses) was 60.6% (95% CI, 26.0–78.5) (Table 2, Figure 2b).

The heterologous booster scheme (two Gam-COVID-Vac/one BNT162b2) effectiveness and no previous infection was 53.7% (95% CI, 13.3–74.7), with the booster shot given within <3 months of the wave’s start. On the other hand, the hybrid immunity (previous infection and two Gam-COVID-Vac/one BNT162b2) effectiveness was higher reaching 90.5% (95% CI, 71.6–97.8) (Table 2, Figure 2b).

The effectiveness of the homologous booster scheme (two BNT162b2/one BNT162b2) and no previous infection was 48.6% (95% CI, −92.2–92.1) and that of previous infection and a homologous BNT162b2 booster was 84.1% (95% CI, 19.6–99.1), with the booster shot given within <3 months of the wave’s start (Table 2, Figure 2b).

These results imply that not only is higher effectiveness reached with hybrid immunity, but longer-lasting immunity is provided after vaccination of individuals who have already contracted COVID-19.

### 4.5. Assessment of Humoral Immunity of Different Vaccination Schemes in Subgroup Analysis

**Figure 3:**
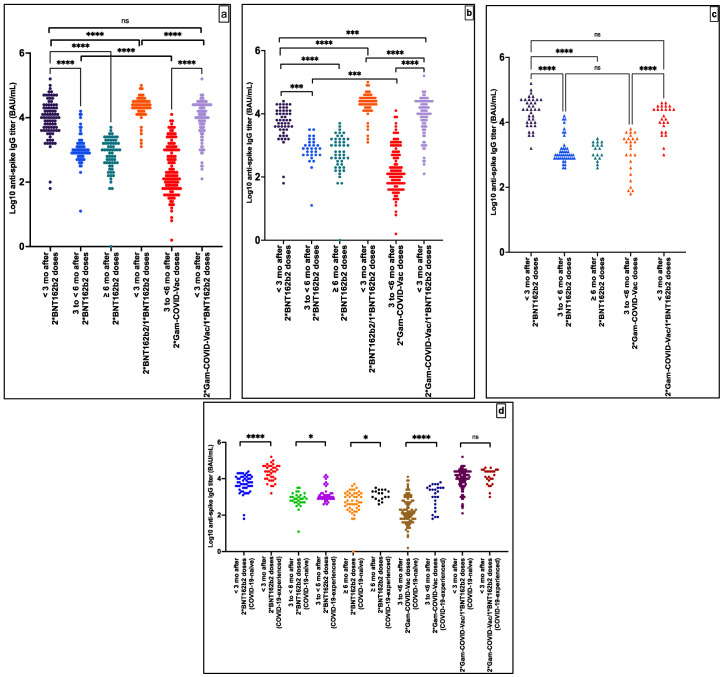
Immune responses against the SARS-CoV-2 spike protein after different vaccination schemes at different time points in subgroup analyses among all participants (**a**), COVID-19-naïve (**b**), COVID-19-experienced (**c**), COVID-19-naïve versus COVID-19-experienced (**d**). N.B.: Results of anti-S-IgG titers are log transformed. Initial measurement results were in U/mL, with the cut-off point defined as 0.80 U/mL to differentiate samples as reactive (≥0.80 U/mL) and non-reactive (<0.80 U/mL) for anti-S-IgG. In all the groups, participants were humorally reactive. ns: (not significant) means *p* > 0.05, * means *p* ≤ 0.05, *** means *p* ≤ 0.001, **** means *p* ≤ 0.0001.

The majority of the recruited participants who received different vaccination schemes in this subgroup analysis were under 50 years of age. There was an almost equal distribution between males and females. Detailed demographic characteristics of the participants recruited for the immunogenicity analysis are presented in Supplementary Table S3. In all the groups, participants were humorally reactive. Results of the anti-S-IgG titers (log transformed) and anti-S-IgG GMT (BAU/mL) in the groups are presented in Figure 3 and Table 3, respectively.

Anti-S-IgG GMT was 5181 BAU/mL (95% CI, 3709–7237) at <3 months after the second BNT162b2 dose among COVID-19-naïve participants. This value significantly decreased by 86% to reach 726 BAU/mL (95% CI, 500 to 1055) at 3 to <6 months after the second BNT162b2 dose and by 90% to reach 536 BAU/mL (95% CI, 351–818) at 6 months after the second dose or thereafter (*p* < 0.0001). Likewise, among all participants (COVID-19-experienced and naïve) who received homologous BNT162b2 vaccination, anti-S-IgG GMT variation with time followed a similar declining pattern among participants (*p* < 0.0001).

Anti-S-IgG GMT was 181 BAU/mL (95% CI, 133 to 248) among COVID-19-naïve participants at 3 to <6 months after the second Gam-COVID-Vac dose, which is significantly less than that of BNT162b2 during the same timeframe (726 BAU/mL, 95% CI, 500–1055) (*p* = 0.02). Likewise, anti-S-IgG GMT was similarly lower among all participants (COVID-19-experienced and naïve) who received homologous Gam-COVID-Vac vaccination (252 BAU/mL, 95% CI, 187–340), when compared to that of homologous BNT162b2 vaccination (1060 BAU/mL, 95% CI, 841–1336) (*p* = 0.01).

Anti-S-IgG GMT significantly increased three-fold to 20,256 BAU/ml (95% CI, 16,244–25,258) among COVID-19-naïve participants, when compared to GMT at <3 months after the second BNT162b2 dose (5181 BAU/mL, 95% CI, 3709–7237, *p* < 0.0001), after a BNT162b2 booster vaccination in the 2*BNT162b2 dose group.

Anti-S-IgG GMT significantly increased 48-fold to 8884 BAU/mL (95% CI, 6966–11,330) among COVID-19-naïve participants (*p* < 0.0001), when compared to GMT at 3 to <6 months after the second Gam-COVID-Vac dose (181 BAU/mL, 95% CI, 133–248, *p* < 0.0001), after a BNT162b2 booster vaccination in the two Gam-COVID-Vac doses group.

A similar increasing anti-S-IgG GMT pattern was recorded when assessing data from all participants (COVID-19-experienced and naïve) (9497 BAU/mL, 95% CI, 7682–11,742) (*p* < 0.0001). Anti-S-IgG GMT of two Gam-COVID-Vac/one BNT162b2 remained significantly lower than that of two BNT162b2/one BNT162b2 (9497 BAU/mL versus 20,256 BAU/mL, respectively) (*p* < 0.0001), yet higher than that at <3 months after the second BNT162b2 dose (5181 BAU/mL, 95% CI, 3709–7237, *p* = 0.001).

### 4.6. Safety of BNT162b2 Booster Vaccination in the Gam-COVID-Vac Primary Vaccination Cohort in Subgroup Analysis

The BNT162b2 booster vaccination was found to be safe and well-tolerated in this group. A total of 77/135 participants (57%) experienced more than one mild-to-moderate AE: generally, muscle or joint pain (33.3%), fever (28.9%), headache (21.5%), and pain at the injection site (15.6%) (Table 4). The AE occurrence was higher among participants below 50 years of age (43/77, 55.8%), when compared to those aged between 50 and 65 years (31/77, 40.3%) (Table 4). There were no potentially life-threatening reactions or hospitalizations due to solicited symptoms within 14 days of the booster vaccination.

## 5. Discussion

### 5.1. Immunogenicity and Effectiveness of Primary Vaccination Schemes with Gam-COVID-Vac and/or BNT162b2

The findings indicate that VE against COVID-19 caused by the Delta variant was lower after two Gam-COVID-Vac doses (with the second dose given within 3 to <6 months prior to the Delta wave) (41.8%), when compared to that after two BNT162b2 (81%) doses (with the second dose given within <3 months of the start of the Delta wave) in this case–control study involving a cohort of employees of the national airline company. These results mirror those of immunogenicity in subgroup analyses, whereby anti-spike IgG levels were higher in the BNT162b2 cohort, when compared to those in the Gam-COVID-Vac cohort. A substantially lower protection was provided by the two-dose BNT162b2 regimen, with the second dose given with 3 to <6 months of the start of the Omicron wave (23.1%), and no protection at all with the two Gam-COVID-Vac regimen given more than 6 months of the Omicron wave’s start, when compared to the Delta variant. Anti-spike IgG levels markedly dropped during these time frames, in parallel to the waning effectiveness.

These data converge with several immunogenic and real-world effectiveness studies globally that documented substantially lower immunity levels and, thus, protection against infection with the Delta and Omicron variants after different COVID-19 vaccines, including BNT162b2 and Gam-COVID-Vac, when compared to previous variants such as Alpha or even the original SARS-CoV-2 strain that was first detected in Wuhan, China [23,24,25,26,27,28,29]. Before the appearance of variants that evaded the effect of the vaccine, serology testing and the stratification of the seroconversion level showed its usefulness to promptly identify high-risk groups who may not develop a viral-neutralizing response, even in the presence of seroconversion, and, therefore, may remain at higher risk of infection, despite vaccination [29].

While the circulating SARS-CoV-2 variant is a major determinant of VE, immunogenicity per se is determined by the type of the vaccine administered, independent of the circulating variant. Nevertheless, the time elapsed since vaccination is a major determinant of the immune response in the vaccinated population during a surge of cases related to a specific variant of concern.

The decrease in immunity and VE can be attributed to several factors. First, humoral immunity was observed to be short-lived and wanes with time when considering the longitudinal dynamics of the serum antibody levels, which was similarly documented in other studies [6,30]. In fact, most of the primary vaccination schemes have already been completed within >3 months of the start of the wave, and even >6 months in developed countries with infections predominantly caused by the Delta variant, whether with BNT162b2 or Gam-COVID-Vac. On the other hand, SARS-CoV-2 variant emergence with multiple substitutions in key antibody epitopes of the S glycoprotein has compromised immunity by partial evasion from neutralizing antibodies, as is the case with the Omicron variant sublineages. Several studies documented immune escape in Omicron among primary and booster vaccine recipients belonging to different platforms that were manufactured based on the ancestral strain [31,32,33,34,35]. In addition, we assume that by the time fast-spreading variants emerge, the number of infected and/or vaccinated individuals would have increased worldwide; thus, inducing herd immunity would in some way affect VE calculation, when considering the unvaccinated COVID-19-naïve individuals as a reference group.

### 5.2. BNT162b2 Booster Immunity and Effectiveness in Participants Previously Vaccinated with Gam-COVID-Vac or BNT162b2

There was a significant multiple-fold increase in anti-S-IgG compared to primary vaccination when a booster BNT162b2 dose was offered to employees previously vaccinated with Gam-COVID-Vac, as shown in our findings. However, the anti-S-IgG GMT produced after a homologous BNT162b2 booster dose was much higher than that produced after a heterologous booster post Gam-COVID-Vac vaccination.

There have been no studies that have checked the heterologous boosting effect of Gam-COVID-Vac-primed individuals with BNT162b2 on the subsequent immune response or VE. Dolzhikova et al. studied the effect of a single-dose Sputnik Light booster given 6–9 months after Gam-COVID-Vac primary vaccination on the level of anti-spike antibodies [36]. Boosting with Sputnik Light significantly increased the level of these antibodies, similar to our findings, whose levels dropped 6 months after primary vaccination [36].

Heterologous boosting of people who previously received primary adenovirus-vectored vaccines, such as ChAdOx1 and Ad26.COV2.S, has already been reported [35,36,37,38]. In the COV-BOOST, a blinded, multicenter, randomized, controlled, phase 2 trial, Munro and colleagues studied the immunogenicity of multiple vaccines from multiple platforms, including mRNA vaccines given after a homologous primary vaccination with either ChAdOx1 or mRNA vaccines [39]. All tested COVID-19 vaccines given as boosters induced a significantly higher immune response after 28 days, compared with their corresponding controls, but significantly lower than the levels reached after the mRNA boosting of the mRNA-primed individuals [39]. Booster vaccines in general enhance waning immunity and expand the breadth of immunity against SARS-CoV-2 variants of concern.

Comparable levels were seen after booster BNT162b2 vaccination in the two Gam-COVID-Vac doses (57%) and two BNT162b2 (64%) doses groups, when the booster dose was given within <3 months of the start of the Omicron wave, regarding effectiveness of the booster schemes.

On the other hand, it is worth noting that the anti-spike antibody levels were much higher after the two BNT162b2/BNT162b2 regimen, in comparison to that after the two Gam-COVID-Vac/BNT162b2 regimen.

The direct positive correlation loss between the IgG levels and effectiveness in the two regimens during the Omicron wave could be explained by the higher affinity of the maturing antibodies triggered by Gam-COVID-Vac vaccination to the newer variants of concern when compared to the ability of BNT162b2 to neutralize these variants.

Investigators analyzed neutralizing antibody response against variants of concern in sera samples of individuals vaccinated with Gam-COVID-Vac and others revaccinated with Sputnik Light in the aforementioned study by Dolzhikova and colleagues [36]. They found that antibodies triggered by Gam-COVID-Vac vaccination after maturation and boosting gained a broader capacity and ability to neutralize the emerging variants of concern [36].

Likewise, another longitudinal study from Argentina showed robust SARS-CoV-2-neutralizing antibodies reduced viral variant escape to neutralization over time, 6 months after Gam-COVID-Vac primary vaccination [40]. Investigators reported that antibodies produced in individuals vaccinated with Gam-COVID-Vac exhibited increased cross-neutralization capacity over time to variants of concern [40].

Our VE and immunogenicity results actually complemented these findings. We hypothesize that the lower anti-spike IgG levels achieved after heterologous boosting among Gam-COVID-Vac recipients, when compared to that after homologous boosting in BNT162b2 recipients, was compensated by a higher neutralizing potency against the Omicron variant based on the aforementioned evidence. This issue led to close VE results achieved in the two schemes, despite the significant differences in anti-spike IgG in favor of homologous BNT162b2 boosting.

Accordingly, heterologous boosting of individuals primed with the Gam-COVID-Vac vaccine with BNT162b2 is effective against the Omicron variant.

Nevertheless, bigger and more structured data are needed to check whether heterologous vaccination and/or heterologous boosting is more effective than homologous regimens against circulating variants of concern.

### 5.3. Longitudinal Dynamics of Effectiveness during the Same Wave

A decline was observed in the protection offered by the two-dose Gam-COVID-Vac vaccination regimen, from 52.3% at the beginning of the wave to 23.7% by the end of the wave, and from 95.1% to 67.5% for the two-dose BNT162b2 scheme, when studying month-by-month variation in VE during the Delta wave. In parallel, as a surrogate marker of humoral immunity, anti-spike IgG levels similarly waned with time during the same wave, with a considerable drop starting at 3 months after vaccination. This decline in both immunogenicity and effectiveness with time has strong implications on the optimal timing of vaccine boosting with the expected next surges in COVID-19 cases caused by variants of concern, as the world is now transitioning to strategies for controlling COVID-19 as an endemic disease. We realize that it takes almost 3–5 months for another wave to occur [41,42,43], upon observing its waves during the past 2 years. Accordingly, the optimal time of a booster dose would be 3–5 months after primary vaccination, depending on the type of primary vaccination and type of circulating variant.

### 5.4. Effect of Hybrid Immunity

Participants with hybrid immunity produced significantly higher anti-spike IgG levels after primary and boosted vaccination schemes, when compared to COVID-19-naïve participants. In the same direction, protection against infection by hybrid immunity was higher than that offered by the vaccine only among COVID-19-naïve individuals.

Several studies have already shown that with all COVID-19 vaccine types, individuals with hybrid immunity have higher neutralizing and spike antibodies compared to COVID-19-naïve primed individuals [44,45]. Chahla and colleagues found that sera from previously infected individuals showed an increased neutralization ability against different variants of concern, and their basal titers had more influence on postvaccination anti-RBD levels than the time elapsed between diagnosis and vaccination [44].

During the Omicron period, national data from Qatar showed that vaccination-induced protection against Omicron infection among persons who had had previous infection [46]. More specifically, hybrid immunity resulting from a previous infection with a pre-Omicron variant and a recent booster vaccination with mRNA vaccines offered the strongest protection [46]. In our cohort, similar results were identified, where the effectiveness of primary BNT162b2 or Gam-COVID-Vac vaccination in COVID-19-experienced individuals was higher than that in COVID-19-naïve individuals. Following a similar pattern, booster BNT162b2 vaccination in individuals previously vaccinated with two doses of BNT162b2 or Gam-COVID-Vac conferred the highest protection against Omicron infection in the COVID-19-experienced group when compared to the COVID-19-naïve group.

We can conclude that the protection of the vaccination among COVID-19-experienced individuals lasted longer when compared to that in COVID-19-naïve individuals, based on the VE calculation in our dataset. These observations give better insights about future vaccination strategies in countries with limited vaccine availability. Accordingly, when putting vaccination strategies in place, the booster dose in COVID-19-experienced individuals could be delayed when compared to the booster dose to be given to COVID-19-naïve individuals, and depending on the type of circulating virus, the delay time can be decided.

### 5.5. Safety

BNT162b2 booster vaccination was found to be safe and well-tolerated 6 months following primary Gam-COVID-Vac vaccination, with regard to safety in our study; no concerns were identified. Reactogenicity was similar to that described in previous evaluations of ChAdOx1 nCov-19, Gam-COVID-Vac, and BNT162b2 vaccines and did not differ between heterologous and homologous boosters [39,47,48].

### 5.6. Importance of Mosaic Vaccination in Epidemic Control and Equity

Disparities in global vaccination progress are large, with low-income countries lagging behind, due to the limited access to vaccines, where only 22% of people in these countries have received at least one dose [49].

These regions of the world contribute to further emergence of variants which fuel public health crises in the developing world and prolong the pandemic. This can only be achieved through equitable global vaccination. A possibility is to propose “Mosaic/Cocktail vaccination” across a population, whereby individuals are vaccinated with a set of qualitatively different vaccines (focused either on different strains or different antigens), which would create a “mosaic” immunity at the population level, leading to a possible improvement in epidemic control [50].

Mosaic vaccination plus heterologous mixing of vaccines may play an important role in ensuring vaccine equity. Making use of different vaccine technologies in cocktail and heterologous protocols would alleviate the high demand on certain vaccines such as the mRNA vaccines. Consequently, the need for “difficult-to-get” vaccines would be cut by half in heterologous primary vaccination or by a third in heterologous boosting [51].

On the other hand, a radical approach to the vaccine-based strategy to control the pandemic would be investing in developing more evolutionarily robust vaccines, either by targeting highly conserved pathogen components (universal vaccines) or by including multiple immunological targets within a single vaccine (multiepitope vaccines).

### 5.7. Limitations

Study limitations include the relatively small sample size when compared to other population-based VE studies in the literature. This was an observational study planned in the middle of the vaccination campaign, where time from vaccination was not well-controlled and not all categories could be compared between the two vaccine platforms. 

The detection of laboratory-confirmed infections was done through SARS-CoV-2 PCR testing of symptomatic employees and others who were asymptomatic during contact tracing. It would have been ideal to undergo systematic screening at several time intervals, so that more asymptomatic infections could have been detected. Nevertheless, this study is based on real-world data in a low-income country with limited resources and facing a socioeconomic crisis. The bias towards including only symptomatic infections rather than all infections was minimized by the vigilant contact tracing system established by the company at the start of the pandemic. Any employee who was in contact with a confirmed case or had a minimal risk of exposure to the virus was screened on several occasions as per US CDC recommendations.

The definition of Delta and Omicron cases was based on epidemiologic evidence rather than genomic testing of the variants in the studied population. The short Omicron period follow-up time was another limitation, in addition to the lack of cellular immunity testing and neutralizing antibody testing in the immunogenicity evaluation.

Another point that merits mention is that our dataset was retrieved from a private company’s database, rather than from a well-structured national registry for COVID-19 infection and vaccination. At the time the study was conducted, such a registry was unavailable in Lebanon, unlike the situation in other middle eastern countries such as Qatar and the United Arab Emirates [19,46,52]. The studied cohort included mostly young and healthy individuals, which is not representative of the Lebanese population regarding age, comorbidities, and socioeconomic status. Nevertheless, our study shed light on the real-world effectiveness of heterologous booster regimens in individuals primed with Gam-COVID-Vac against the vaccine-tolerant Omicron variant.

## 6. Conclusions

In this case–control cohort study involving national airline company employees, waning antibody levels were shown with time after primary BNT162b2 or Gam-COVID-Vac vaccination, accompanied by a parallel decline in effectiveness during the phases where the Delta and Omicron variants predominated in Lebanon.

Boosting with BNT162b2 after primary BNT162b2 or Gam-COVID-Vac vaccination is safe. It induces a substantial increase in humoral immunity, with a comparable effectiveness against Omicron infections in both groups.

We also showed that hybrid immunity offers longer protection against COVID-19 when compared to vaccination only.

Time since vaccination and vaccine evasion by new variants of concern are the main players in real-world effectiveness.

Our effectiveness and immunogenicity findings mainly during the Omicron period prove the need for modified vaccination strategies, emphasizing the importance of mosaic or cocktail vaccination among the same population, or even developing second-generation vaccines with epitopes that match the mutated variants, or finding epitopes that are more stable with time and are less subject to mutations.

Primary and booster COVID-19 vaccination campaigns should be reinforced mainly at the beginning of each wave, since vaccination becomes less efficient as the wave progresses, and knowing that we are still far from controlling the pandemic.

## Figures and Tables

**Table 1:** Effectiveness of the available COVID-19 vaccination schemes during the Delta wave.

	Effectiveness against Delta Infection				
	Cases		Controls		Effectiveness % (95% Confidence Interval)
	Vaccinated	Unvaccinated	Vaccinated	Unvaccinated	
2 × Gam-COVID-Vac doses (2nd dose given 3 to <6 months prior to start of wave) (All participants)	175	23	2698	193	41.8 (5.6 to 62.6)
Month 1 (August 2021)	36	6	2772	210	52.3 (−27.4 to 78.7)
Month 2 (September 2021)	20	2	2752	208	22.4 (−85 to 77.6)
Month 3 (October 2021)	9	3	2743	205	76.9 (−4.8 to 93.2)
Month 4 (November 2021)	37	5	2706	200	44.1 (−64.4 to 76.3)
Month 5 (December 2021)	73	7	2633	193	23.7 (−84.7 to 62.8)
2 × Gam-COVID-Vac doses (2nd dose given 3 to <6 months prior to start of wave) (COVID-19-naïve)	68	7	2043	151	20.1 (−94.7 to 61.6)
Month 1 (August 2021)	0	0	2060	158	-
Month 2 (September 2021)	0	0	2060	158	-
Month 3 (October 2021)	0	0	2060	158	-
Month 4 (November 2021)	0	0	2060	158	-
Month 5 (December 2021)	68	7	1992	151	20.1 (−94.7 to 61.6)
2 × Gam-COVID-Vac doses (2nd dose given 3 to <6 months prior to start of wave) (COVID-19-experienced)	107	7	655	151	82.6 (44.4 to 94.9)
Month 1 (August 2021)	36	0	712	158	-
Month 2 (September 2021)	20	0	692	158	-
Month 3 (October 2021)	9	0	683	158	-
Month 4 (November 2021)	37	0	646	158	-
Month 5 (December 2021)	5	7	641	151	82.6 (44.4 to 94.9)
2 × BNT162b2 doses (2nd dose given <3 months prior to start of wave) (All participants)	33	23	1383	193	81.0 (66.3 to 89.2)
Month 1 (August 2021)	2	6	1414	210	95.1 (78.4 to 99.3)
Month 2 (September 2021)	1	2	1413	208	92.2 (16.3 to 99.6)
Month 3 (October 2021)	4	3	1409	205	80.6 (15 to 95.8)
Month 4 (November 2021)	12	5	1397	200	72.7 (12.5 to 90.1)
Month 5 (December 2021)	14	7	1383	193	67.5 (12.8 to 87.2)
2 ×BNT162b2 doses (2nd dose given <3 months prior to start of wave) (COVID-19-naïve)	11	7	841	151	71.8 (22.3 to 89.1)
Month 1 (August 2021)	0	0	852	158	-
Month 2 (September 2021)	0	0	852	158	-
Month 3 (October 2021)	0	0	852	158	-
Month 4 (November 2021)	0	0	852	158	-
Month 5 (December 2021)	11	7	841	151	71.8 (22.3 to 89.1)
2 × BNT162b2 doses (2nd dose given <3 months prior to start of wave) (COVID-19-experienced)	22	7	542	151	88.1 (56.5 to 97.5)
Month 1 (August 2021)	2	0	562	158	-
Month 2 (September 2021)	1	0	561	158	-
Month 3 (October 2021)	4	0	557	158	-
Month 4 (November 2021)	12	0	545	158	-
Month 5 (December 2021)	3	7	542	151	88.1 (56.5 to 97.5)

**Table 2:** Effectiveness of the available COVID-19 vaccination schemes during the Omicron wave.

	Effectiveness against Omicron Infection				
	Cases		Controls		Effectiveness % (95% Confidence Interval)
	Vaccinated	Unvaccinated	Vaccinated	Unvaccinated	
2 × Gam-COVID-Vac doses (2nd dose given >6 months prior to start of wave)					
All participants	315	22	1755	181	−54.1 (−151 to 0.84)
COVID-19-naïve	245	19	1205	127	−46 (−149.7 to 10.1)
COVID-19-experienced	70	19	550	127	18.8 (−43.8 to 52.2)
2 × BNT162b2 doses (2nd dose given 3 to <6 months prior to start of wave)					
All participants	109	22	1251	181	23.1 (−28.7 to 52.1)
COVID-19-naïve	77	19	742	127	21.9 (−38.9 to 54.1)
COVID-19-experienced	32	19	509	127	60.6 (26.0 to 78.5)
2 × Gam-COVID-Vac/1 × BNT162b2 doses (booster dose given <3 months prior to start of wave)					
All participants	34	22	731	181	57.0 (23.2 to 75.5)
COVID-19-naïve	32	19	529	127	53.7 (13.3 to 74.7)
COVID-19-experienced	2	19	202	127	90.5 (71.6 to 97.8)
2 × BNT162b2/1 × BNT162b2 doses (booster dose given <3 months prior to start of wave)					
All participants	3	22	68	181	63.7 (−9.1 to 91.6)
COVID-19-naïve	2	19	26	127	48.6 (−92.2 to 92.1)
COVID-19-experienced	1	19	42	127	84.1 (19.6 to 99.1)

**Table 3:** Immunogenicity results of different vaccination schemes at different time points.

	All Participants	COVID-19-Naïve	COVID-19-Experienced
<3 months after 2 × BNT162b2 doses (N = 87 individuals)	9473 (7156–12,542)	5181 (3709–7237)	21,413 (15,170–30,227)
3 to <6 months after 2 × BNT162b2 doses (N = 75 individuals)	1060 (841–1336)	726 (500–1055)	1363 (1028–1808)
≥6 months after 2 × BNT162b2 doses (N = 68 individuals)	676 (485–941)	536 (351–818)	1283 (938–1756)
<3 months after 2 × BNT162b2/1 × BNT162b2 doses (N = 60 individuals)	20,256 (16,244–25,258)	20,256 (16,244–25,258)	-
3 to <6 months after 2 × Gam-COVID-Vac (N = 135 individuals)	252 (187–340)	181 (133–248)	1070 (581–1971)
<3 months after 2 × Gam-COVID-Vac/1 × BNT162b2 doses (N = 135 individuals)	9497 (7682–11,742)	8884 (6966–11,330)	12,741 (8367–19,401)

Abbreviations: BAU = Binding Antibody Unit.

**Table 4:** Adverse events reported after BNT162b2 booster vaccination in the Gam-COVID-Vac primary vaccination cohort in subgroup analysis stratified by age and gender.

	Any Adverse Event	Muscle or Joint Pain	Fever	Headache	Pain at Injection Site	Chills	Lethargy	Nausea	Dyspnea	Vomiting	Diarrhea	Abdominal Pain
Total (N = 135)	77 (57.0%)	45 (33.3%)	39 (28.9%)	29 (21.5%)	21 (15.6%)	19 (14.1%)	7 (5.2%)	5 (3.7%)	2(1.5%)	1 (0.7%)	1 (0.7%)	1 (0.7%)
Age (years)												
<50	43 (55.8%)	26 (57.8%)	25 (64.1%)	17 (58.6%)	10 (47.6%)	13 (68.4%)	5 (71.4%)	3 (60%)	1 (50%)	1 (100%)	0	1 (100%)
50–65	31 (40.3%)	16 (35.6%)	14 (35.9%)	11 (37.9%)	10 (47.6%)	6 (31.6%)	2 (28.6%)	2 (40%)	1 (50%)	0	1 (100%)	0
>65	3 (3.9%)	3 (6.7%)	0	1 (3.9%)	1 (4.8%)	0	0	0	0	0	0	0
Gender												
Male	40 (3.9%)	21 (46.7%)	18 (46.2%)	12 (41.4%)	12 (57.1%)	8 (42.1%)	4 (57.1%)	0	1 (50%)	0	1 (100%)	0
Female	37 (51.9%)	24 (53.3%)	21 (53.8%)	17 (58.6%)	9 (42.9%)	11 (57.9%)	3 (42.9%)	5 (100%)	1 (50%)	1 (100%)	0	1 (100%)

## Data Availability

The data that support the findings of this study will be shared on reasonable request to the corresponding author.

## Supplementary Materials

The following supporting information can be downloaded at: https://www.mdpi.com/article/10.3390/vaccines10101596/s1, Table S1: Demographic Characteristics of the Participants Used to Estimate Vaccine Effectiveness of Vaccination Schemes including BNT162b2 or Gam-COVID-Vac during the Delta Wave, Table S2: Demographic Characteristics of the Participants Used to Estimate Vaccine Effectiveness of Vaccination Schemes including BNT162b2 or Gam-COVID-Vac during the Omicron Wave, Table S3: Demographic data of Participants in Immunogenicity Subgroup Analyses.
