# Supplementary material for: Immunogenicity and Effectiveness of Primary and Booster Vaccine Combination Strategies during Periods of SARS-CoV-2 Delta and Omicron Variants
Source: Vaccines (Basel). 2022 Sep 22;10(10):1596. doi: 10.3390/vaccines10101596 (PMC9607825; doi:10.3390/vaccines10101596)
Supplement: Supplementary file 1 [file vaccines-10-01596-s001.zip › vaccines-1894912-supplementary.pdf]

Table S1. Demographic characteristics of the participants used to estimate effectiveness of vaccination schemes including BNT162b2 or Gam-COVID-Vac during the Delta wave.

	No vaccination						2 Gam-COVID-Vac doses (2nd dose given 3 to < 6 months prior to start of the wave)						2 BNT162b2 doses (2nd dose given < 3 months prior to start of the wave)					
	All participants (N=216)		COVID-19-naïve (N=158)		COVID-19-experi-enced (N=58)		All participants (N=2875)		COVID-19-naïve (N=2113)		COVID-19-experi-enced (N=762)		All participants (N=1416)		COVID-19-naïve (N=852)		COVID-19-experi-enced (N=564)	
	Cases	Controls	Cases	Controls	Cases	Controls	Cases	Controls	Cases	Controls	Cases	Controls	Cases	Controls	Cases	Controls	Cases	Controls
**Delta waive (August to December 2021) (Total number)**	**23**	**193**	**7**	**151**	**16**	**42**	**177**	**2698**	**70**	**2043**	**107**	**655**	**33**	**1383**	**11**	**841**	**22**	**542**
**Age (years)**																		
<50	22	144	7	113	15	31	149	1897	59	1381	90	516	32	1095	11	655	21	440
50-65	1	40	0	31	1	9	27	763	10	630	17	133	1	287	0	185	1	102
>65	0	9	0	7	0	2	1	38	1	32	0	6	0	1	0	1	0	0
**Gender**																		
Male	17	127	3	99	14	28	109	1939	44	1482	65	457	23	1095	5	681	18	414
Female	6	66	4	52	2	14	68	759	26	561	42	198	10	288	6	160	4	128
**August 2021 (Total number)**	**6**	**210**	**0**	**158**	**6**	**52**	**36**	**2772**	**0**	**2060**	**36**	**712**	**2**	**1414**	**0**	**852**	**2**	**562**
**Age (years)**																		
<50	6	160	0	120	6	40	30	1988	0	1421	30	567	2	1127	0	666	2	461
50-65	0	41	0	31	0	10	6	751	0	611	6	140	0	286	0	185	0	101
>65	0	9	0	7	0	2	0	33	0	28	0	5	0	1	0	1	0	0
**Gender**																		
Male	6	138	0	102	6	36	18	1977	0	1484	18	493	2	1116	0	686	2	430
Female	0	72	0	56	0	16	18	795	0	576	18	219	0	298	0	166	0	132
**September 2021 (Total number)**	**2**	**208**	**0**	**158**	**2**	**50**	**20**	**2752**	**0**	**2060**	**20**	**692**	**1**	**1413**	**0**	**852**	**1**	**561**
**Age (years)**																		
<50	1	159	0	120	1	39	15	1973	0	1421	15	552	1	1124	0	666	1	458
50-65	1	40	0	31	1	9	5	746	0	611	5	135	0	286	0	185	0	101
>65	0	9	0	7	0	2	0	33	0	28	0	5	0	1	0	1	0	0
**Gender**																		
Male	1	137	0	102	1	35	12	1965	0	1484	12	481	1	1115	0	686	1	429
Female	1	71	0	56	1	15	8	787	0	576	8	211	0	298	0	166	0	132
**October 2021 (Total number)**	**3**	**205**	**0**	**158**	**3**	**47**	**9**	**2743**	**0**	**2060**	**9**	**683**	**4**	**1409**	**0**	**852**	**4**	**557**

**Age (years)**																		
<50	3	156	0	120	3	36	7	1966	0	1421	7	545	4	1120	0	666	4	454
50-65	0	40	0	31	0	9	2	744	0	611	2	133	0	286	0	185	0	101
>65	0	9	0	7	0	2	0	33	0	28	0	5	0	1	0	1	0	0
**Gender**																		
Male	2	135	0	102	2	33	6	1959	0	1484	6	475	3	1112	0	686	3	426
Female	1	70	0	56	1	14	3	784	0	576	3	208	1	297	0	166	1	131
**November 2021 (Total number)**	**5**	**200**	**0**	**158**	**5**	**42**	**37**	**2706**	**0**	**2060**	**37**	**646**	**12**	**1397**	**0**	**852**	**12**	**545**
**Age (years)**																		
<50	5	151	0	120	5	31	34	1932	0	1421	34	511	11	1109	0	666	11	443
50-65	0	0	0	31	0	9	3	741	0	611	3	130	1	285	0	185	1	100
>65	0	0	0	7	0	2	0	33	0	28	0	5	0	1	0	1	0	0
**Gender**																		
Male	5	130	0	102	5	28	26	1933	0	1484	26	449	11	1101	0	686	11	415
Female	0	70	0	56	0	14	11	733	0	576	11	157	1	296	0	166	1	130
**December 2021 (Total number)**	**7**	**193**	**7**	**151**	**0**	**42**	**73**	**2633**	**68**	**1992**	**5**	**641**	**14**	**1383**	**11**	**841**	**3**	**542**
**Age (years)**																		
<50	7	193	7	113	0	31	62	1870	58	1363	4	507	14	1095	11	655	3	440
50-65	0	0	0	31	0	9	10	731	9	602	1	129	0	285	0	185	0	100
>65	0	0	0	7	0	2	1	32	1	27	0	5	0	1	0	1	0	0
**Gender**																		
Male	3	127	3	99	0	28	45	1888	43	1441	2	447	6	1095	5	681	1	414
Female	4	66	4	52	0	14	28	745	25	551	3	194	8	288	6	160	2	128

Table S2. Demographic characteristics of the participants used to estimate effectiveness of vaccination schemes including BNT162b2 or Gam-COVID-Vac during the Omicron wave.

	**No vaccination**						**2 Gam-COVID-Vac doses (2nd dose given > 6 months prior to start of wave)**						**2 BNT162b2 doses (2nd dose given 3 to < 6 months prior to start of wave)**						**2xGam-COVID-Vac/1xBNT162b2 doses (3rd dose given < 3 months prior to start of wave)**						**2xBNT162b2/1xBNT162b2 doses (3rd dose given < 3 months prior to start of wave)**					
	**All participants (N=203)**		**COVID-19-naïve (N=146)**		**COVID-19-experienced (N=57)**		**All participants (N=2070)**		**COVID-19-naïve (N=1450)**		**COVID-19-experienced (N=620)**		**All participants (N=1360)**		**COVID-19-naïve (N=819)**		**COVID-19-experienced (N=541)**		**All participants (N=765)**		**COVID-19-naïve (N=561)**		**COVID-19-experienced (N=204)**		**All participants (N=71)**		**COVID-19-naïve (N=28)**		**COVID-19-experienced (N=43)**	
	Cases	Controls	Cases	Controls	Cases	Controls	Cases	Controls	Cases	Controls	Cases	Controls	Cases	Controls	Cases	Controls	Cases	Controls	Cases	Controls	Cases	Controls	Cases	Controls	Cases	Controls	Cases	Controls	Cases	Controls
**Omicron wave (January-February 2022) (Total number)**	**22**	**181**	**19**	**127**	**3**	**54**	**315**	**1755**	**245**	**1205**	**70**	**550**	**109**	**1251**	**77**	**742**	**32**	**509**	**34**	**731**	**32**	**529**	**2**	**202**	**3**	**68**	**2**	**26**	**1**	**42**
**Age (years)**																														
<50	22	132	19	89	3	43	269	1284	208	836	61	448	93	1003	64	578	29	425	26	455	24	306	2	149	2	40	2	16	0	24
50-65	0	40	0	31	0	9	42	454	34	354	8	100	16	245	13	163	3	82	8	263	8	214	0	49	0	28	0	10	0	18
>65	0	9	0	7	0	2	4	17	3	15	1	2	0	3	0	1	0	2	0	13	0	9	0	4	1	0	0	0	1	0
**Gender**																														
Male	12	121	10	86	2	35	195	1287	152	12	43	418	74	1007	52	616	22	391	16	516	15	375	1	141	1	50	0	18	1	32
Female	10	60	9	41	1	19	120	468	93	1193	27	202	35	244	25	126	10	118	18	215	17	154	1	61	2	18	2	8	0	10

Table S3. Demographic data of participants in immunogenicity subgroup analyses.

	**< 3 months after 2xBNT162b2 doses (N=87)**	**3 to < 6 months after 2xBNT162b2 doses (N=75)**	**≥ 6 months after 2xBNT162b2 doses (N=68)**	**< 3 months after 2xBNT162b2/1xBNT162b2 doses (N=60)**	**3 to <6 months after 2xGam-COVID-Vac (N=135)**	**< 3 months after 2xGam-COVID-Vac/1xBNT162b2 doses (N=135)**
**Age (years)**						
<50	80 (92.0%)	64 (85.3%)	32 (47.1%)	36 (60%)	67 (49.6%)	67 (49.6%)
50-65	7 (8.0%)	10 (13.3%)	23 (33.8%)	17 (28.3%)	61 (45.2%)	61 (45.2%)
>65	0	1 (1.3%)	13 (19.1%)	7 (11.7%)	7 (5.2%)	7 (5.2%)
**Gender**						
Male	59 (67.8%)	43 (57.3%)	37 (54.4%)	35 (58.3%)	76 (56.3%)	76 (56.3%)
Female	28 (32.2%)	32 (42.7%)	31 (45.6%)	25 (41.7%)	59 (43.7%)	59 (43.7%)
